# Improved detection of prostate cancer using a magneto-nanosensor assay for serum circulating autoantibodies

**DOI:** 10.1371/journal.pone.0221051

**Published:** 2019-08-12

**Authors:** Lingyun Xu, Jung-Rok Lee, Shiying Hao, Xuefeng Bruce Ling, James D. Brooks, Shan X. Wang, Sanjiv Sam Gambhir

**Affiliations:** 1 Department of Radiology, Molecular Imaging Program at Stanford, Bio-X Program, Stanford University School of Medicine, Stanford, California, United States of America; 2 Division of Mechanical and Biomedical Engineering, Ewha Womans University, Seoul, South Korea; 3 Clinical and Translational Research Program, Betty Irene Moore Children's Heart Center, Lucile Packard Children’s Hospital, Palo Alto, California, United States of America; 4 Departments of Surgery, Stanford University, Stanford, California, United States of America; 5 Department of Urology, Stanford University School of Medicine, Stanford, California, United States of America; 6 Department of Materials Science & Engineering, Stanford University, Stanford, California, United States of America; 7 Department of Electrical Engineering, Stanford University, Stanford, California, United States of America; 8 Department of Radiology, Stanford University School of Medicine, Stanford, California, United States of America; 9 Department of Bioengineering, Stanford University, Stanford, California, United States of America; The Ohio State University, UNITED STATES

## Abstract

**Purpose:**

To develop a magneto-nanosensor (MNS) based multiplex assay to measure protein and autoantibody biomarkers from human serum for prostate cancer (CaP) diagnosis.

**Materials and methods:**

A 4-panel MNS autoantibody assay and a MNS protein assay were developed and optimized in our labs. Using these assays, serum concentration of six biomarkers including prostate-specific antigen (PSA) protein, free/total PSA ratio, as well as four autoantibodies against Parkinson disease 7 (PARK7), TAR DNA-binding protein 43 (TARDBP), Talin 1 (TLN1), and Caldesmon 1 (CALD1) and were analyzed. Human serum samples from 99 patients (50 with non-cancer and 49 with clinically localized CaP) were evaluated.

**Results:**

The MNS assay showed excellent performance characteristics and no cross-reactivity. All autoantibody assays showed a statistically significant difference between CaP and non-cancer samples except for PARK7. The most significant difference was the combination of the four autoantibodies as a panel in addition to the free/total PSA ratio. This combination had the highest area under the curve (AUC)– 0.916 in ROC analysis.

**Conclusions:**

Our results suggest that this autoantibody panel along with PSA and free PSA have potential to segregate patients without cancer from those with prostate cancer with higher sensitivity and specificity than PSA alone.

## Introduction

Prostate cancer (CaP) constitutes a major health burden in men. In 2017, it is estimated that over 160,000 men were diagnosed with CaP, and it remains the second leading cause of cancer-related deaths in men [1]. Because of its prolonged natural history and potential curability while localized, CaP has been regarded as the optimal cancer for screening approaches as a means of reducing mortality. Starting in the late 1980s, serum Prostate Specific Antigen (PSA) testing began to be used for CaP screening and became widespread in the U.S. over the next decade. Several recent randomized trials have placed PSA testing in context. While the European Randomized Study of Screening for Prostate Cancer (ERSPC) showed small but significant improvements in CaP mortality with PSA screening, the Prostate Lung Colon Ovarian (PLCO) screening study showed PSA testing led to many unnecessary biopsies that caused significant morbidities including pain, sepsis, bleeding, and overdiagnosis. [2–4]

Analysis of molecular forms of PSA, such as free PSA, complexed PSA and pro-PSA have slightly improved the sensitivity and specificity of PSA testing, thereby eliminating some unnecessary biopsies [5]. However, there is a significant need to find new biomarkers to complement or replace PSA. There is clearly an important need for new reliable diagnostic and prognostic biomarkers. Recently, several lines of evidence suggest that panels of biomarkers such as microRNAs [6, 7], mRNA [8], SNP[9] and the 4Kscore test [10], can better identify men with CaP, compared to any single-biomarker assay.

The work presented in this paper focused on developing a novel panel of serum biomarkers to be used in testing to distinguish non-cancer patients from CaP patients. We are interested in noninvasive serum biomarkers due to their ease of collection and low cost for large screening. Recent studies have shown the potential of autoantibody screening in different types of cancer, e.g., colorectal cancer [11], pancreatic cancer [12], cervical cancer [13], and lung cancer [14]. The onset of autoantibodies also allows a more detailed look into molecular processes in early disease development.

We selected 4 candidate autoantibodies against 4 proteins (TARDBP, PARK7, Talin 1, TLN1, and CALD1) that have been previously shown to be associated with prostate cancer. We paired them with total PSA (includes both complexed and uncomplexed forms) and free-PSA (uncomplexed to chaperone proteins) to develop a panel for improved prostate cancer diagnosis [15].

In addition, new diagnostic tools that are robust, sensitive, and accurate are also needed for affordable and rapid screening of this panel. In this study we report the design, development, validation, and initial application of a magneto nanosensor (MNS) based multiplex assay that can simultaneously detect biomarkers. MNS is a magnetic nanoparticle-based analytical device that employs the effect of giant magnetoresistance (GMR) to produce electrical signals proportional to the concentration of the analytes [16]. Upon linking magnetic nanoparticles to the bound analytes on the sensor surface, the resistance of the magneto-nanosensors is changed due to the stray magnetic field from the nanoparticles [17]. An array of MNSs has been fabricated to perform multiplexed assays with a single chip [18, 19] and demonstrated to detect radiation-exposure biomarkers [20, 21], autoantibodies [22], and cancer biomarkers [23]. With this technique, the PSA and autoantibody levels in 99 CaP and benign prostatic hypertrophy (BPH) patient serum samples were analyzed, and we found that this panel is capable of differentiating CaP from BPH with an ROC AUC of 0.916.

## Materials and methods

### Recombinant proteins & antibodies

Optimal matched pairs including capture recombinant protein and detection anti-human IgG antibody, and the monoclonal antibody standards were obtained from the following vendors:

CALD1: The anti-CALD1 (R&D Systems; MAB7569) was a mouse monoclonal antibody. The capture human recombinant protein (H00000800-P01) was obtained from Abnova.

PARK7: The anti-PARK7 (Abnova; MAB1076) was a mouse monoclonal antibody. The capture human recombinant protein (H00011315-P01) was obtained from Abnova.

TARDBP: The anti-TARDBP (Abnova; H00023435-M01) was a mouse monoclonal antibody. The capture human recombinant protein (H00023435-Q07) was obtained from Abnova.

TLN1: The anti-TLN1 (Abnova; H0007094-M05) was a mouse monoclonal antibody. The capture human recombinant protein (H00007094-P01) was obtained from Abnova.

Detecting IgG: The anti-human IgG biotinylated antibody (109-065-098) was obtained from Jackson Immuno Research.

For total PSA and free PSA assay, we used native human PSA prepared in our laboratory as the standard [24]. The monoclonal antibodies were obtained from Meridian:

Total PSA: the capture antibody (Meridian; M66280M) and the detection antibody (Meridian; M66276M) were both mouse monoclonal IgG.

Free PSA: the capture antibody (Meridian; M86806M) and the detection Ab (Meridian; M66276M) were both mouse monoclonal IgG.

### Assay reagents

Poly allylamine hydrochloride (Polyscience), poly ethylene-alt-maleic anhydride (Aldrich), phosphate buffered saline (PBS) (Invitrogen), 1-ethyl-3-(3-dimethylaminopropyl) carbodiimide hydrochloride (EDC) (Thermo scientific), N-hydroxysuccinimide (NHS) (Aldrich), bovine serum albumin (BSA) (Aldrich), Tween 20 (Aldrich), and streptavidin-coated MicroBeads (Miltenyi, 130-048-101) were used as received, and without further purification.

### Magneto-nanosensor assay

The MNS chip was designed by MagArray Inc. Eighty MNSs were fabricated on each chip by a previously reported method [19]. The chip has a dimension of 10 × 12 mm, and an array of 10 × 8 MNSs is located in the middle of the chip with a spacing of 400 μm between neighboring sensors. The sensor chip surface was treated with NHS-EDC for immobilization of the capture proteins as previously described [25]. Briefly, the sensor chip was sequentially washed with acetone, methanol, and isopropanol. After cleaned using oxygen plasma, the chip was treated with poly(allylamine hydrochloride) and poly(ethylene-alt-maleic anhydride).

A robotic spotter (Scienion, sciFlexarrayer) was then used to deposit capture recombinant protein (0.5 mg/ml in PBS) onto the surface of individual MNSs. Each sample had four replicates. 1% BSA was placed over 6 sensors to serve as biological negative controls. Eight reference sensors were covered with a thicker passivation layer to measure electrical background signals. Finally, the sensor chip was stored in a humidity chamber at 4°C overnight before use. After washing the sensor chip surface with a washing buffer (0.1% BSA and 0.05% Tween 20 in PBS), the surface was blocked with 1% BSA for 1 hour. Then, the chip was washed again and the 1:100 diluted serum samples were spotted on the sensors using the robotic spotter. After incubating the MNS chip in a humidity chamber at 4°C overnight, the chip was washed with the washing buffer, and a solution of anti-human IgG detection antibodies at a concentration of 5 μg/ml was added. Following 1-hour incubation with the detection antibodies, the chip was washed again before the MNS chip was inserted into the reader station and the baseline signals from the sensors were measured. After addition of 50 μl streptavidin-coated magnetic nanoparticles, the changes in the magnetoresistive (MR) ratio was recorded by the station [16]. To measure the MR ratio, an oscillating magnetic field is externally applied to modulate electrical signals from the sensors while currents at another frequency are applied to the sensors. As the magnetic nanoparticles bind to the sensor surface, the MR ratio increases. The plateaued signals were taken as MNS signals, and the average signal of electrical reference sensors were subtracted from them for further analyses.

### Samples

De-identified serum samples from men with CaP and without prostate cancer were obtained under Stanford University Internal Review Board (IRB) approved protocol. In this study, 99 samples (50 men without cancer on 2 separate biopsies and 49 pre-operative samples from men with CaP) (Table 1) were selected. Based on surgical pathology of the CaP samples we assessed a full spectrum of cancer Gleason scores: one patient was Gleason score 6; 38 were Gleason score 7, and 10 were Gleason score 9. Samples were diluted in PBS (1:100) for testing.

**Table 1:** Patient information.

	CaP	BPH
n	49	50
Mean Age ± SEM	64.1 ± 1.0	66.6 ± 1.1
Mean Total PSA ± SEM (ng/ml)	16.21 ± 1.6	7.7 1 ± 0.7
Gleason Score (≤ 6)	1	
Gleason Score (7–8)	38	
Gleason Score (9)	10	
Mean Tumor Volume (mm^2)^	8.49 ± 0.68	
PARK7 (ppm)	1469 ± 30	1454 ± 39
TARDBP1 (ppm)	1216 ± 30	1477 ± 37
TLN1 (ppm)	1380 ± 35	1542 ± 48
CALD1 (ppm)	1190 ± 29	1602 ± 63
Free/Total PSA	0.022 ± 0.005	0.072 ± 0.019

### Statistics

Univariate analysis was performed to calculate the odds ratios and p-values (likelihood ratio test) for each biomarker studied. Based on the analysis results, samples were randomly split into 5 equal-sized subgroups, with numbers of cases and controls remaining in balance in each subgroup. For each biomarker panel, a linear discrimination analysis (LDA) model was developed to explore the relationship between the disease and the biomarkers. The independent variables were biomarker values (i.e., predictors of the model), and the dependent variable was a binary value (0 for non-cancer controls, and 1 for CaP cases). The LDA algorithm produced a score for each sample, describing the probability it would be a ‘case’. The higher the score, the higher was the probability that the corresponding sample would have CaP. The model was trained with four subgroups (so-called training sub-cohort) and tested on the rest one subgroup (so-called testing sub-cohort) in turn as a 5-fold cross-validation process, in order to avoid an over-fitting problem. Results on the testing sub-cohorts were integrated for each biomarker panel. An ROC curve was plotted to show the binary classification characteristic of our models. C-statistics and 95% CI were calculated to measure the model performance in differentiating cases from controls.

## Results

### MNS assay development

**Fig 1:**
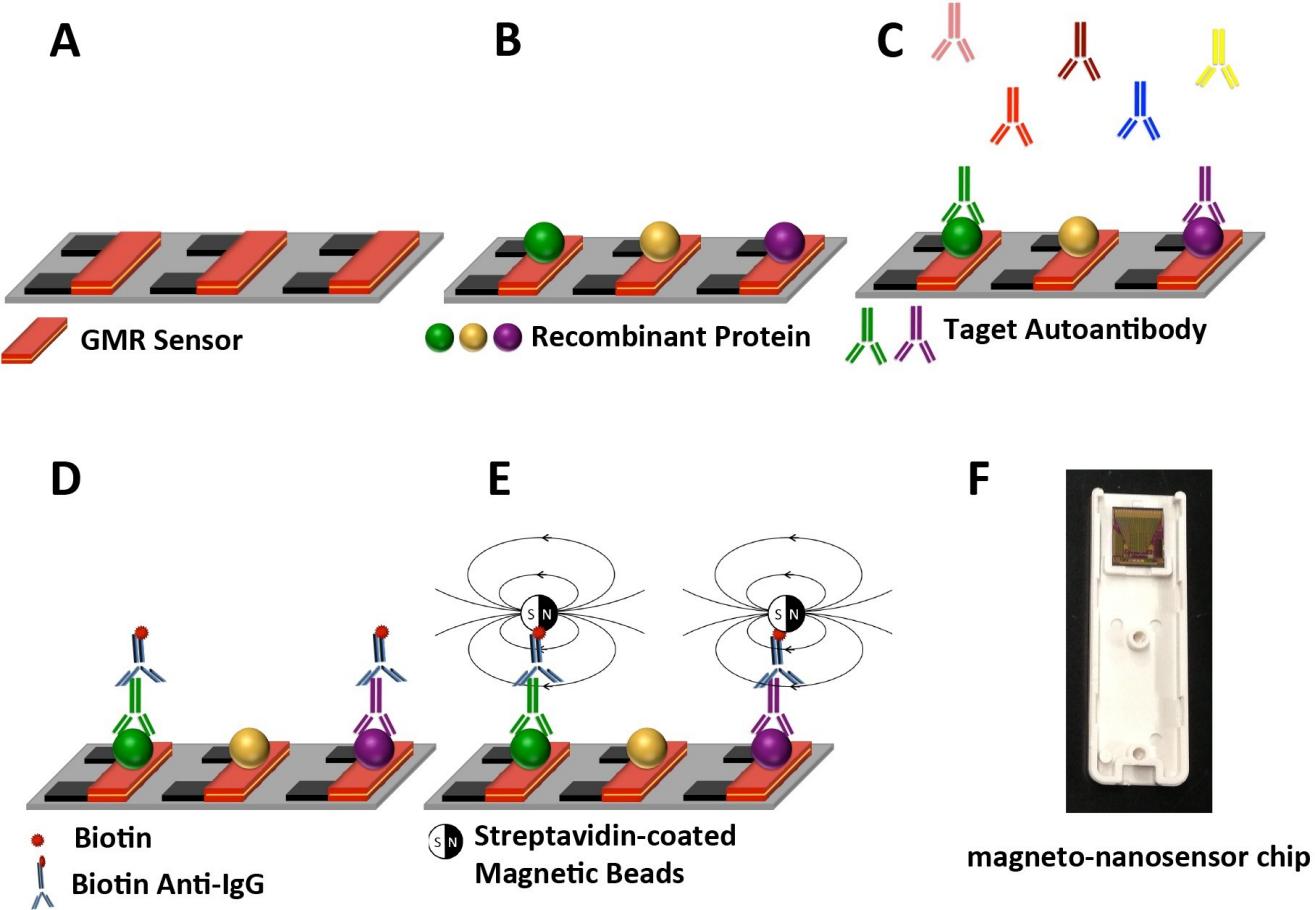
Schematic of multiplexed magnetic autoantibody immunoassay. (A) Each MNS chip contains an array of 80 individual giant magnetoresistor sensors (shown in orange). (B) The commercially available, recombinant proteins, specific to their respective autoantibodies which may or may not be present in a given patients sample [16], are immobilized on the nanosensors as capture protein. Three different colors represent three different proteins. (C) The serum samples are robotically spotted onto those sensors and the target autoantibodies (if they exist in a given patient sample) are captured by the immobilized protein. (D) After washing away the unbound autoantibodies, biotinylated anti-human IgG antibodies are added and reacted with each of the bound autoantibodies as the detection antibody. (E) Finally, the streptavidin-coated magnetic nanoparticles (not shown in scale) are added. The binding of the nanoparticles to the biotinylated anti-human IgG antibodies disturbs local magnetic fields, and induces the changes in the resistance of the MNS at a given location which allows one to determine which autoantibody must be present. (F) Real photo of a MNS chip (10 cm X 12 cm).

We developed a multiplex immunoassay magneto-nanosensor chip to investigate the capacity of a panel of four CaP-related autoantibodies, and PSA and free/total PSA ratio to differentiate CaP from non-cancer cases. Each MNS chip contained 72 effective sensors along with 8 electrical reference sensors. This chip allowed us to test multiple biomarkers for many patient samples with a single chip. Fig 1 shows the schematic of the autoantibody immunoassay: multiple recombinant proteins were immobilized onto different nanosensor surfaces using a robotic spotter. Followed by PBS wash, serum samples were prepared at 1:100 dilution and spotted onto those sensors. Target autoantibodies in solution that bind to the capture protein were tagged with biotinylated anti-human IgG antibody. When an external oscillating magnetic field was applied, stray fields induced from the bound streptavidin-coated magnetic nanoparticles changed the sensor resistance compared to the baseline resistance without the nanoparticles. Using a custom designed electrical read-out system, the change in resistance was then recorded as a measure of the number of bound magnetic nanoparticles, and thus autoantibody concentration.

For free and total PSA detection, a well-characterized sandwich-based MNS protein assay was performed with a pair of antibodies (capture and detection) surrounding each analyte as previously described [23].

### Assay characterization

**Fig 2:**
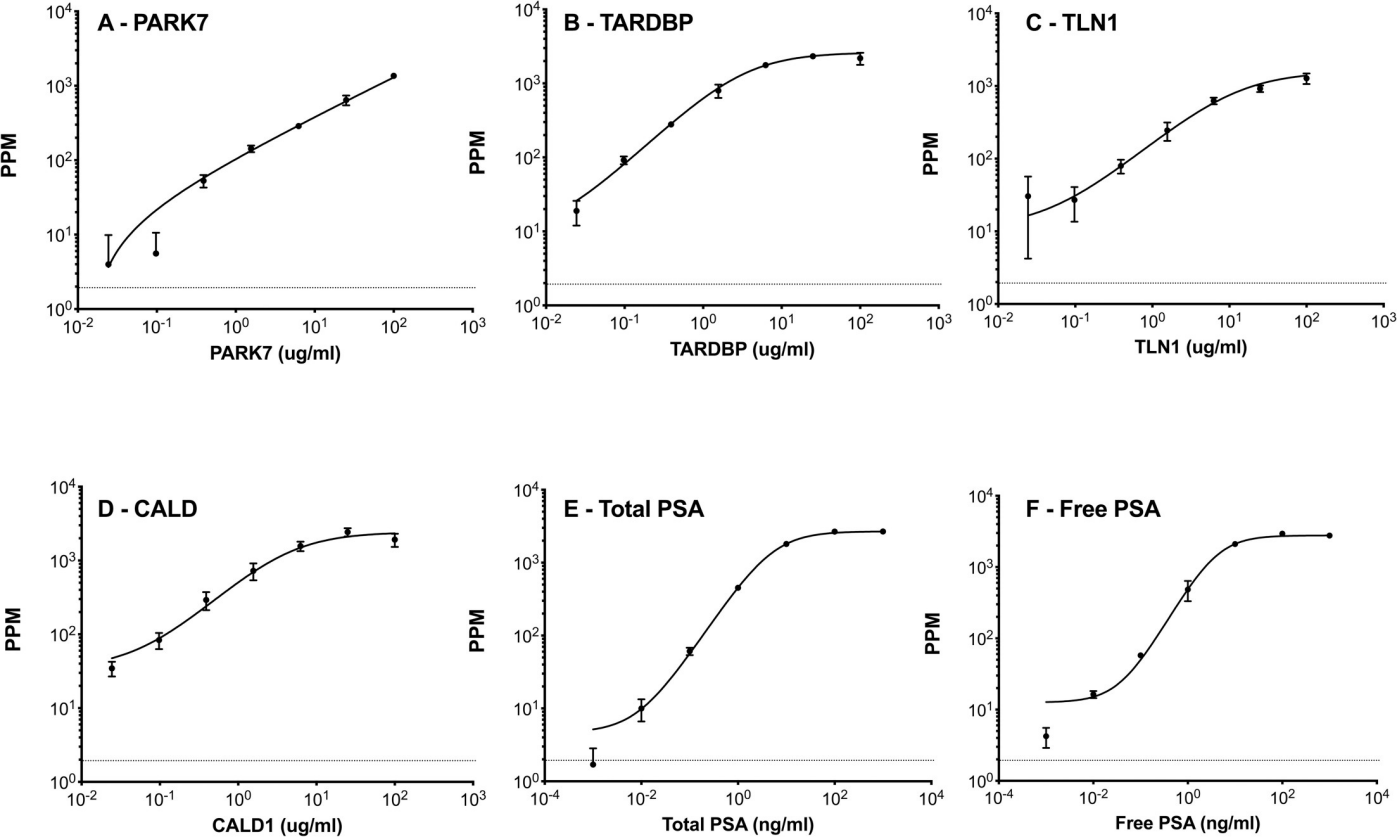
Titration curves acquired from multiplexed MNS immunoassay. Eight point titration curves were generated using MNS chips. For four autoantibodies (A) PARK7; (B) TARDBP; (C) TLN1 and (D) CALD1, the commercial monoclonal antibodies were used as standards. For (E) total PSA and (F) free PSA, the purified PSA protein was used as standard. Each data point represents the average of four replicates. The dotted lines are background level measured without any target analyte in solution. Error bars indicate ± 1 standard deviation. The data points of each assay fit with a four parameter logistic curve.

We generated titration curves to calibrate the measurement of the biomarkers in human serum by spiking various concentrations of the standards into PBS-based buffer solution (Fig 2). Commercial monoclonal antibodies were diluted in PBS containing 0.1% BSA at 20 ng/ml to 200 μg/ml as the standards for the autoantibody assays. Purified human PSA (0.01 ng/ml to 1000 ng/ml) was used as the standard for the free and total PSA assays. Eight point titration curves were created using multiplexed magneto-nanosensor assays. The standard curves all had a linear dynamic range of more than three orders of magnitude. The human serum samples were diluted 100 times in PBS to ensure that each biomarker readout signal would be within the linear range of the standard curve.

**Fig 3:**
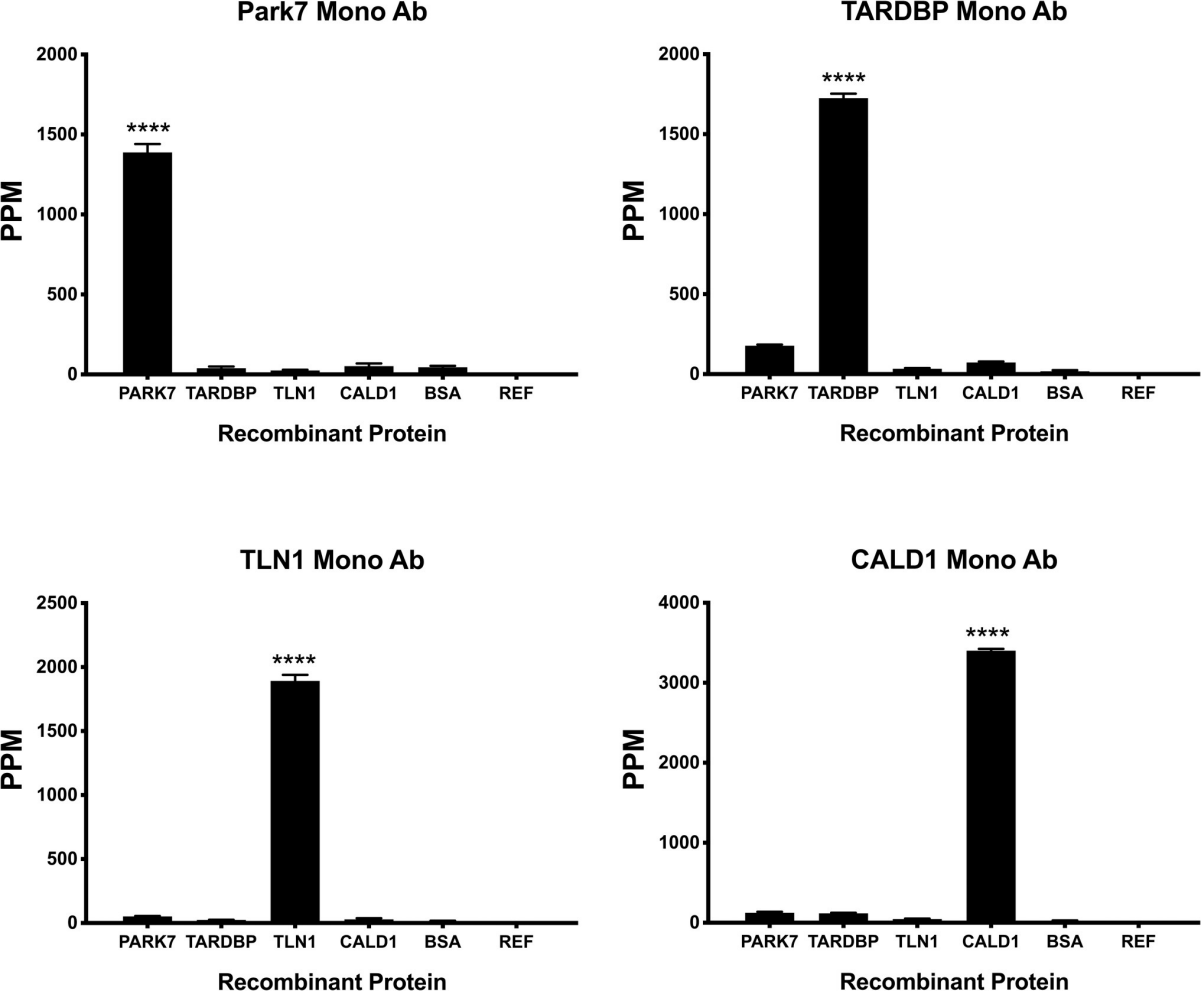
The cross-reactivity test for autoantibody immunoassay. Four recombinant capture proteins PARK7, TARDBP, TLN1 and CALD1 were immobilized on a chip alone with 1% BSA as a negative control (BSA). Commercial monoclonal antibodies were added to the chip. The specific binding of the antibody to the capture protein was measured and each antibody was shown to display no cross reactivity with all other proteins. The specific binding signal was significantly above non-specific signal with p< 0.001 using ANOVA test. The MNS chip background level was measured by a reference sensor (REF). Each column represents the average signal of four replicates. Error bars indicate ± 1 standard deviation.

For the next step, to validate whether there is nonspecific binding between different autoantibody biomarkers in multiplex MNS assays, the cross-reactivity between each biomarker was further examined. The recombinant proteins of four biomarkers were immobilized on a chip, and a high concentration of one kind of monoclonal antibody was added to the chip. Fig 3 shows the highly specific binding of standard antibody to the target protein. As can be seen, the non-target protein had very weak or no binding to the added antibody in each test.

### Biomarker concentration in clinical samples

In our cohort (Table 1, CaP n = 49; Non-cancer n = 50), 99 patient serum samples from the Stanford University Urology department were analyzed using MNS chips. The serum levels of four autoantibodies and the ratio of free/total PSA were measured and the mean value of those biomarkers for the cases of Non-cancer and CaP cases are shown in Table 1.

Odds ratios were calculated, and the biomarkers that showed significant difference between Non-cancer and CaP were free/total PSA (p = 0.003), TARDBP (p<0.001), TLN1 (p = 0.006) and CALD1 (p<0.001) (Table 2).

**Table 2:** *P* value, odds ratio and its 0.95 confidence interval of each biomarker, to discriminate disease from BPH control samples.

Biomarker	Log OR, 95%CI	*P* Value
Total PSA (ng/ml)	0.15374 (0.07099–0.25484)	<0.001
Free PSA (ng/ml)	0.61234 (-0.4431–2.318)	0.278
Ratio (Free vs. Total PSA)	-13.52642 (-29.72827 - -3/13651)	0.003
PARK7 (ppm)	0.00024 (-0.00136–0.00185)	0.771
TARDBP (ppm)	-0.00488 (-0.00726 - -0.00285)	<0.001
TLN1(ppm)	-0.00192 (-0.00348 - -0.00052)	0.006
CALD (ppm)	-0.00419 (-0.0062 - -0.00252)	<0.001

**Fig 4:**
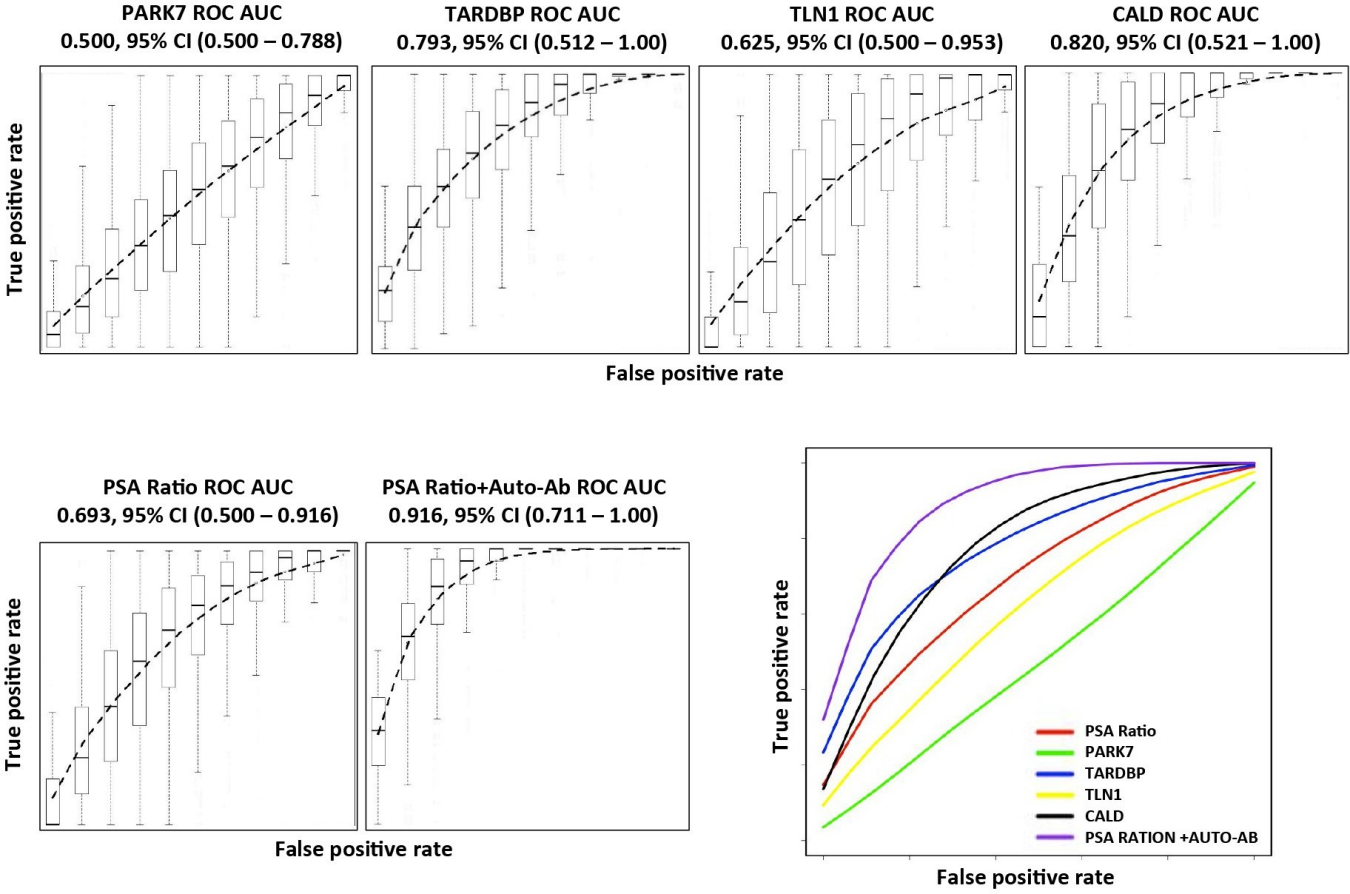
The receiver operating characteristic (ROC) curves of biomarkers. ROC curves for PARK7, TARDBP, TLN1, CALD1 and free/total PSA ratio. Samples were from 50 BPH and 49 CaP (Gleason Score 6–9 patients). The linear discriminant analysis-derived (LDA) prediction scores for each sample were used to construct an ROC curve; 500 testing data sets, generated by bootstrapping, from the biomarker panel were used to derive estimates of standard errors and confidence intervals for our ROC analysis. 5-fold cross validation was integrated into the performance analysis. The plotted ROC curve is the vertical average of the 500 bootstrapping runs, and the box and whisker plots show the vertical spread around the average. The mean and 0.95 confidence interval of ROC AUC values were calculated from 500 bootstrapping results. The biomarker panel consists of free/total PSA ratio, PARK7, TARDBP, TLN1 and CALD1.

Receiver operating characteristic (ROC) curves were created using 5-fold cross validation [20] and the area under the curve (AUC) for all biomarkers was calculated. Those AUCs for the biomarkers individually are shown in Fig 4. The AUC value for free/Total PSA was 0.693, which is within the typical value range in the literature [26–30]. Interestingly, the autoantibodies all displayed significant AUCs with the exception of PARK7, Which had an AUC value of 0.51 compared to other biomarkers, whose AUC ranged from 0.625–0.820.

Combination of the four autoantibodies as a panel along with the free/total PSA ratio resulted in a dramatic shift of the AUC to the left with a significant improvement of the AUC value to 0.916. All biomarkers contributed to the improved AUC, implying that they each provided independent diagnostic information.

**Fig 5:**
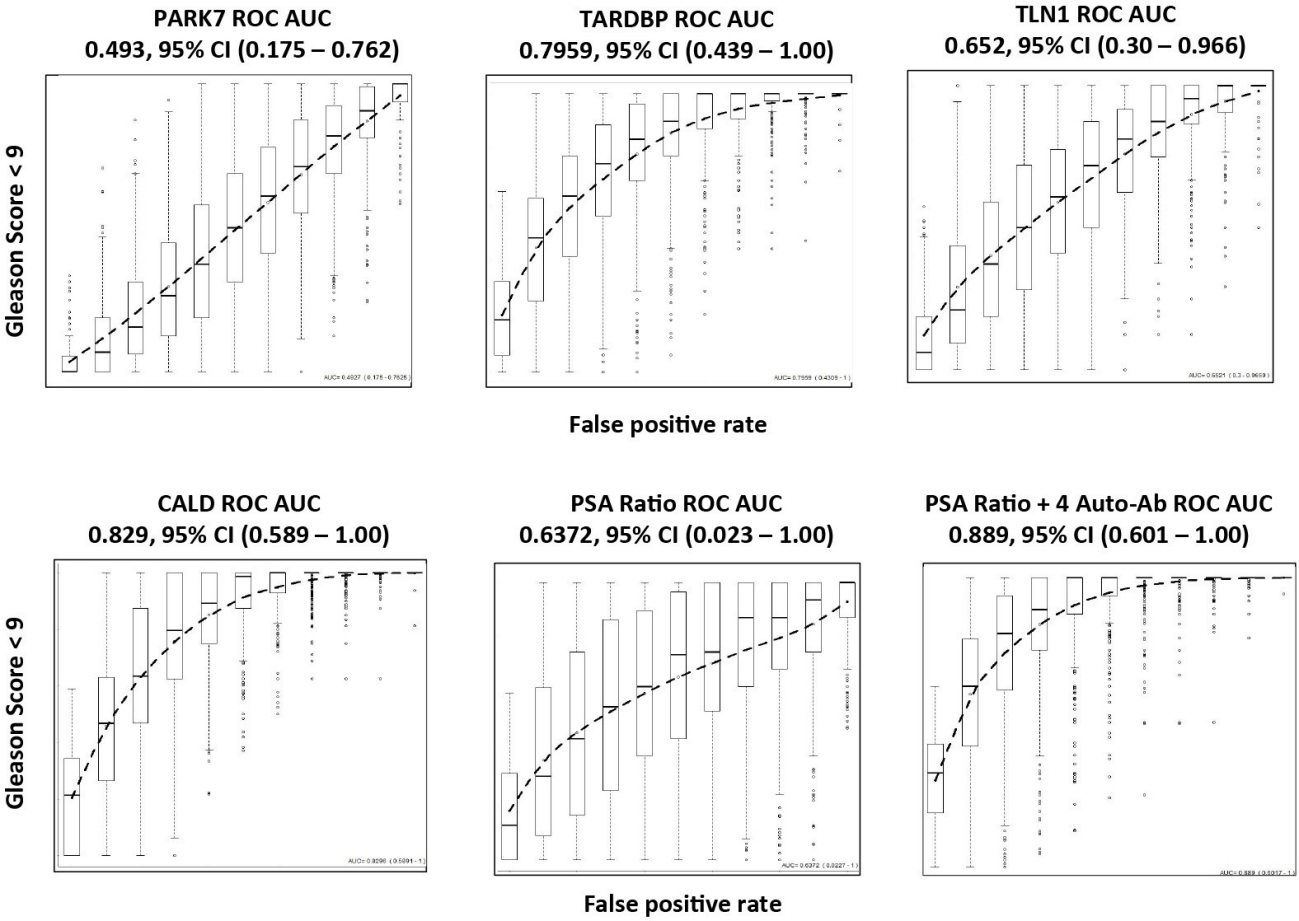
The ROC curves of biomarkers using lower Gleason Score patients. ROC curves for PARK7, TARDBP, TLN1, CALD1 and free/total PSA ratio. Samples used were from 50 BPH and 39 CaP patients. The 39 CaP patients consisted of one patient with Gleason Score 6 and 38 patients with a Gleason Score of 7.

We further studied whether the performance of the biomarkers was influenced by the inclusion of patients with high-grade cancer. We removed the 10 samples with Gleason Score of 9, and re-analyzed the remaining 39 CaP compared with 50 non-cancer samples using the same statistical methods (Fig 5). Interestingly, the lower Gleason Score group generated similar ROC curves and AUC values for all 4 autoantibodies as seen when all 49 patients were included. For the panel that included the 4 autoantibodies and the free/total PSA ratio, the AUC value decreased slightly from 0.916 to 0.889. However this was still better than the free/total PSA alone (AUC = 0.6372).

Thus, using our MNS data, we constructed a panel of four autoantibodies along with the PSA ratio. This panel demonstrated a feasible assay to segregate patients with BPH from prostate cancer.

## Discussion

In this study, we have described the development of a 4-plex magneto-nanosensor autoantibody assay for the simultaneous measuring of autoantibodies. The assay shows excellent performance characteristics and no significant cross-reactivity. Multiplexing offers several distinct advantages over single-plex assays. The most obvious is that our assay enables researchers to extract more data from a limit amount of sample as a compare to a single-plex assay like ELISA. The typical ELISA assay requires 100 microliter sample for one protein detection. By comparison, MNS assay just require 10 microliter or less sample for up to 80 analytes. The multiplex capability also allows for easy addition of other biomarkers, especially autoantibody biomarkers, into the existing panel. Our current panel can successfully discriminate non-cancer cases from CaP. With addition of new biomarkers, this multiplex clinical diagnostic tool may be able to potentially be used for better early cancer detection, and also distinguishing different cancer stages or monitoring the therapeutic response. The performance of the MNS for detecting autoantibodies suggests this platform could be used in other contexts where detection of autoantibodies is useful in diagnosis. Furthermore, our preliminary data strongly suggest that autoantibody panels could be combined with serum proteins in developing future diagnostic tests in prostate cancer and other cancer types.

Unlike ELISA-based protein assays that require significant time and expense to find a good matched antibody pair with suitable performance characteristics for robust assay construction, the autoantibody assay only needs to find a suitable single recombinant protein as the capture reagent. Using a single detection reagent (anti-human IgG antibody) for all autoantibodies also minimizes the cross-reactivity between analyte channels. However, we did have a few promising candidates that failed in the assay development stage due to poor specificity, indicating that there are still challenges in using autoantibodies. The MNS platform also speeds assay development and were able to complete optimization of the assays for all four autoantibodies in less than one month to finish. The quick candidate selection and assay optimizing provide a very efficient assay development procedure.

We describe a unique serum biomarker panel composed of autoantibodies and free and total PSA that potentially improves the detection of men with CaP from men without cancer. Furthermore, we describe simultaneous measurement of these autoantibodies on a novel magneto-nanosensor platform that allows rapid detection on a single chip. Combining these biomarkers significantly improves the performance of PSA with ROC AUC curve improvements from approximately 0.7 to 0.9. This significant shift, if validated, could significantly improve prostate cancer screening by eliminating unnecessary biopsies in men who have an elevated PSA for other reasons, such as BPH. Although the AUC of the assay with PARK7 alone is 0.5, When PARK7 is removed from the panel, the AUC drops to 0.8428 (S1 Fig), indicating the four autoantibodies plus PSA ratio is still the best. We also report that this same combination panel can be used to distinguish the lower Gleason Score cancer patients from BPH patients. While promising, this panel will need additional testing and validation on independent and larger patient sets.

Auto-antibodies in cancer are believed to arise from re-expression of dormant proteins or mutations that create novel epitopes that are then exposed to the immune system. One advantages of using circulating autoantibodies as biomarkers is their relatively high concentration in blood and long half-life. Previous publications in other cancer types have shown the potential value of a panel of autoantibodies as detection biomarkers [31, 32]. Autoantibody panels have been tested as potential biomarkers for the early detection of esophageal squamous cell carcinoma [33], colorectal cancer [11], pancreatic cancer [12], cervical cancer [13], gastric cancer [30, 34] and lung cancer [14]. Previous publications indicate that all four of the biomarkers tested in the current work (PAK7, TARDBP, TLN1 and CALD1) are highly expressed in prostate tumor cells as compared with non-cancerous prostate tissues [35–40]. They also play important roles in hepatocellular carcinoma [41], Amyotrophic lateral sclerosis [42], clear cell renal cell carcinoma [43], breast cancer [44], pancreatic cancer [45], bladder cancer [46].prostate cancer patients [37, 38, 40]. Our study further supports those observations by showing a high level of autoantibodies in the serum of CaP patients. However, the molecular mechanism of higher autoantibody levels in BPH as compared with CaP remains unknown. Our hypothesis is both BPH and CaP patients produce fairly constant autoantibodies in blood, but the CaP patients may produce more antigens. Then more antigens from CaP may actually lead to less free autoantibodies in circulation. Since our chip immobilized antigen can only capture free autoantibodies, our assay would thus report a lower level of autoantibody in CaP cases.

To further validate the performance of the MNS assay, additional studies, including increasing the patients sample size to several hundred in each group and adding a normal age matched control group to current cohort, are required. The MNS assay shows potential as a diagnostic tool and should be validated with further study.

## Supporting information

S1 Fig The ROC curves of biomarkers using free/total PSA ratio, TARDBP, TLN1 and CALD1. ROC curve for the biomarker panel consists of free/total PSA ratio, TARDBP, TLN1 and CALD1. Samples used were from 50 BPH and 49 CaP patients.
(TIF)

## Abbreviations

AUC: area under the curve
BPH: benign prostatic hypertrophy
CALD1: caldesmon 1
CaP: prostate cancer
LDA: linear discrimination analysis
MNS: magneto-nanosensor
MR: magnetoresistive
PARK7: parkinson disease 7
PSA: prostate-specific antigen
ROC: receiver operating characteristic
TARDBP: TAR DNA-binding protein 43
TLN1: talin 1
