# Supplementary figures and images for: Improved detection of prostate cancer using a magneto-nanosensor assay for serum circulating autoantibodies
Source: PLoS One. 2019 Aug 12;14(8):e0221051. doi: 10.1371/journal.pone.0221051 (PMC6690541; doi:10.1371/journal.pone.0221051)

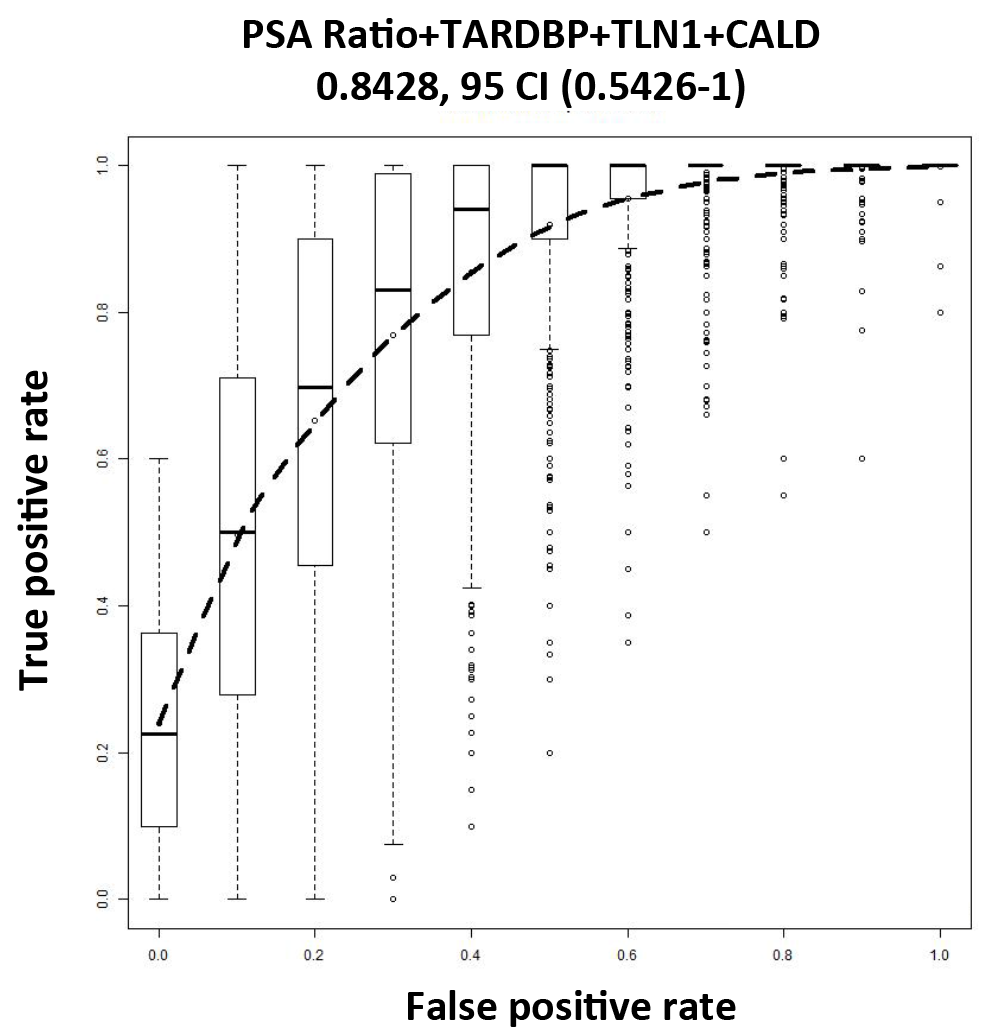

Supplement: S1 Fig — ROC curve for the biomarker panel consists of free/total PSA ratio, TARDBP, TLN1 and CALD1. Samples used were from 50 BPH and 49 CaP patients. (TIF) [file pone.0221051.s001.tif]
